# Supplementary material for: Structural basis for different types of hetero-tetrameric light-harvesting complexes in a diatom PSII-FCPII supercomplex
Source: Nat Commun. 2022 Apr 1;13:1764. doi: 10.1038/s41467-022-29294-5 (PMC8976053; doi:10.1038/s41467-022-29294-5)
Supplement: Supplementary file 1 — Supplementary Information [file 41467_2022_29294_MOESM1_ESM.pdf]

Supplementary Information

Structural basis for different types of hetero-tetrameric light-harvesting complexes in a diatom PSII-FCPII supercomplex

Ryo Nagao[1,†,*], Koji Kato[1,†], Minoru Kumazawa[2], Kentaro Ifuku[3], Makio Yokono[4], Takehiro Suzuki[5], Naoshi Dohmae[5], Fusamichi Akita[1], Seiji Akimoto[6,*], Naoyuki Miyazaki[7,*], and Jian-Ren Shen[1,*]

[1]*Research Institute for Interdisciplinary Science and Graduate School of Natural Science and Technology, Okayama University, Okayama 700-8530, Japan*
[2]*Graduate School of Biostudies, Kyoto University, Kyoto 606-8502, Japan*
[3]*Graduate School of Agriculture, Kyoto University, Kyoto 606-8502, Japan*
[4] *Institute of Low Temperature Science, Hokkaido University, Hokkaido 060-0819, Japan*
[5]*Biomolecular Characterization Unit, RIKEN Center for Sustainable Resource Science, Saitama 351-0198, Japan*
[6]*Graduate School of Science, Kobe University, Hyogo 657-8501, Japan*
[7]*Life Science Center for Survival Dynamics, Tsukuba Advanced Research Alliance (TARA), University of Tsukuba, Ibaraki 305-8577, Japan.*

[†]These authors contributed equally to this work.

*Corresponding Authors:
Ryo Nagao, E-mail: nagaoryo@okayama-u.ac.jp
Seiji Akimoto, E-mail: akimoto@hawk.kobe-u.ac.jp
Naoyuki Miyazaki, E-mail: naomiyazaki@gmail.com
Jian-Ren Shen, E-mail: shen@cc.okayama-u.ac.jp

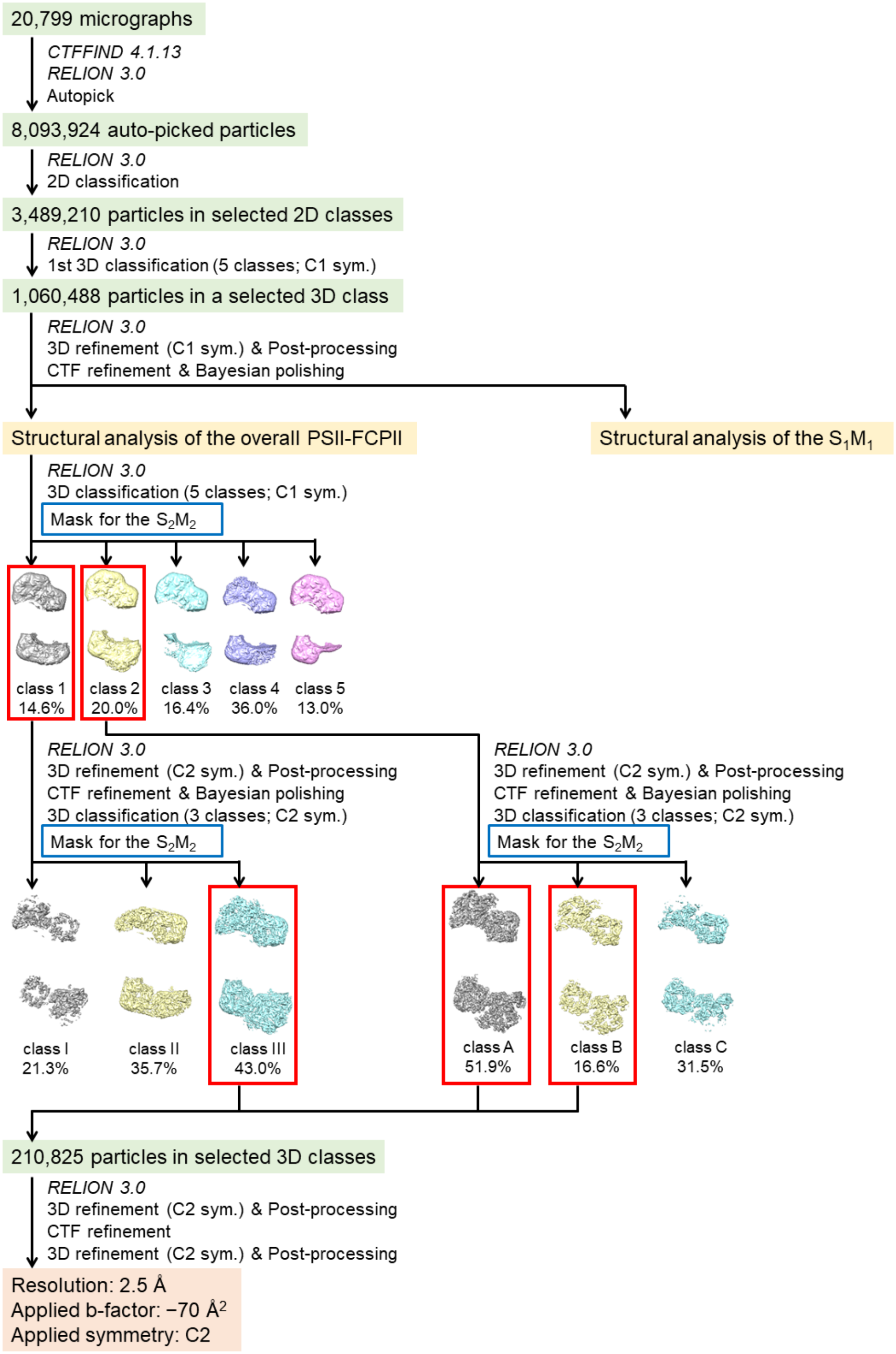
20,799 micrographs
CTFFIND 4.1.13
RELION 3.0
Autopick
8,093,924 auto-picked particles
RELION 3.0
2D classification
3,489,210 particles in selected 2D classes
RELION 3.0
1st 3D classification (5 classes; C1 sym.)
1,060,488 particles in a selected 3D class
RELION 3.0
3D refinement (C1 sym.) & Post-processing
CTF refinement & Bayesian polishing
Structural analysis of the overall PSII-FCPII
Structural analysis of the S1M1
RELION 3.0
3D classification (5 classes; C1 sym.)
Mask for the S2M2
class 1 14.6%
class 2 20.0%
class 3 16.4%
class 4 36.0%
class 5 13.0%
RELION 3.0
3D refinement (C2 sym.) & Post-processing
CTF refinement & Bayesian polishing
3D classification (3 classes; C2 sym.)
Mask for the S2M2
RELION 3.0
3D refinement (C2 sym.) & Post-processing
CTF refinement & Bayesian polishing
3D classification (3 classes; C2 sym.)
Mask for the S2M2
class I 21.3%
class II 35.7%
class III 43.0%
class A 51.9%
class B 16.6%
class C 31.5%
210,825 particles in selected 3D classes
RELION 3.0
3D refinement (C2 sym.) & Post-processing
CTF refinement
3D refinement (C2 sym.) & Post-processing
Resolution: 2.5 Å
Applied b-factor: −70 Å2
Applied symmetry: C2

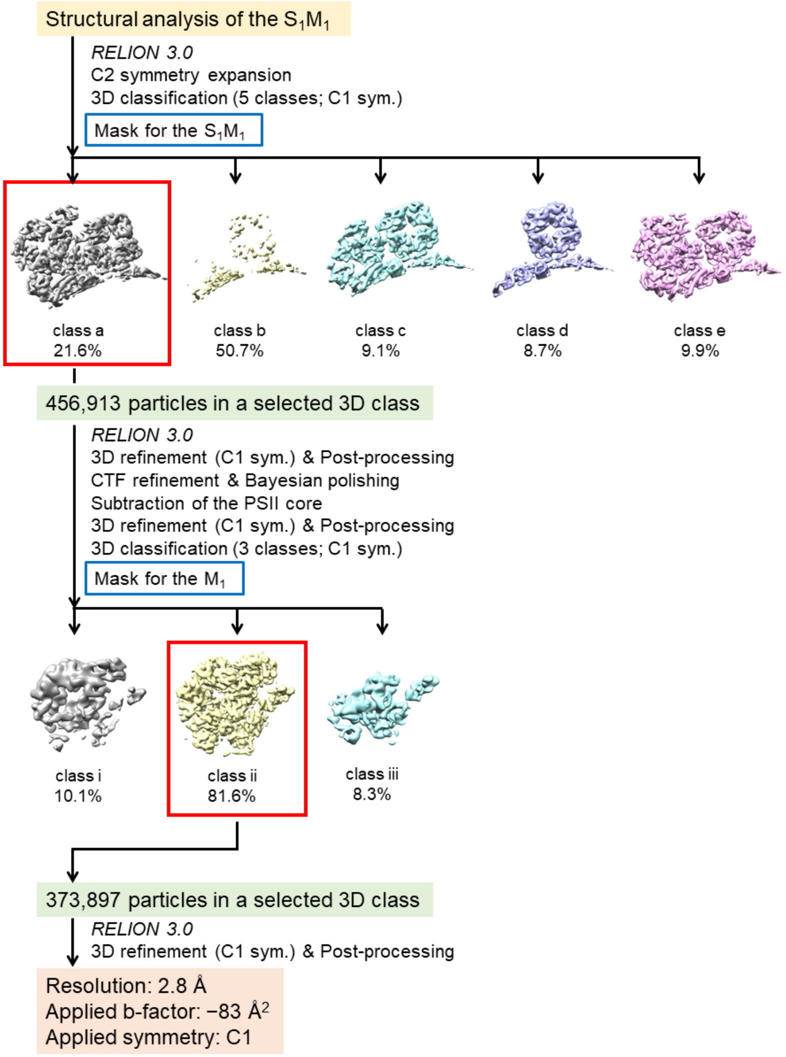

Supplementary Fig. 1. Cryo-EM data processing of the PSII-FCPII supercomplex. Schematic flowchart showing the classification of PSII-FCPII. The overall PSII-FCPII ($C_2S_2M_2$) structure was reconstructed at 2.5 Å resolution from 210,825 particles, whereas the S_1M_1 structure of FCPII from the subtracted particles was reconstructed at 2.8 Å resolution from 373,897 particles. See Methods section for more details.

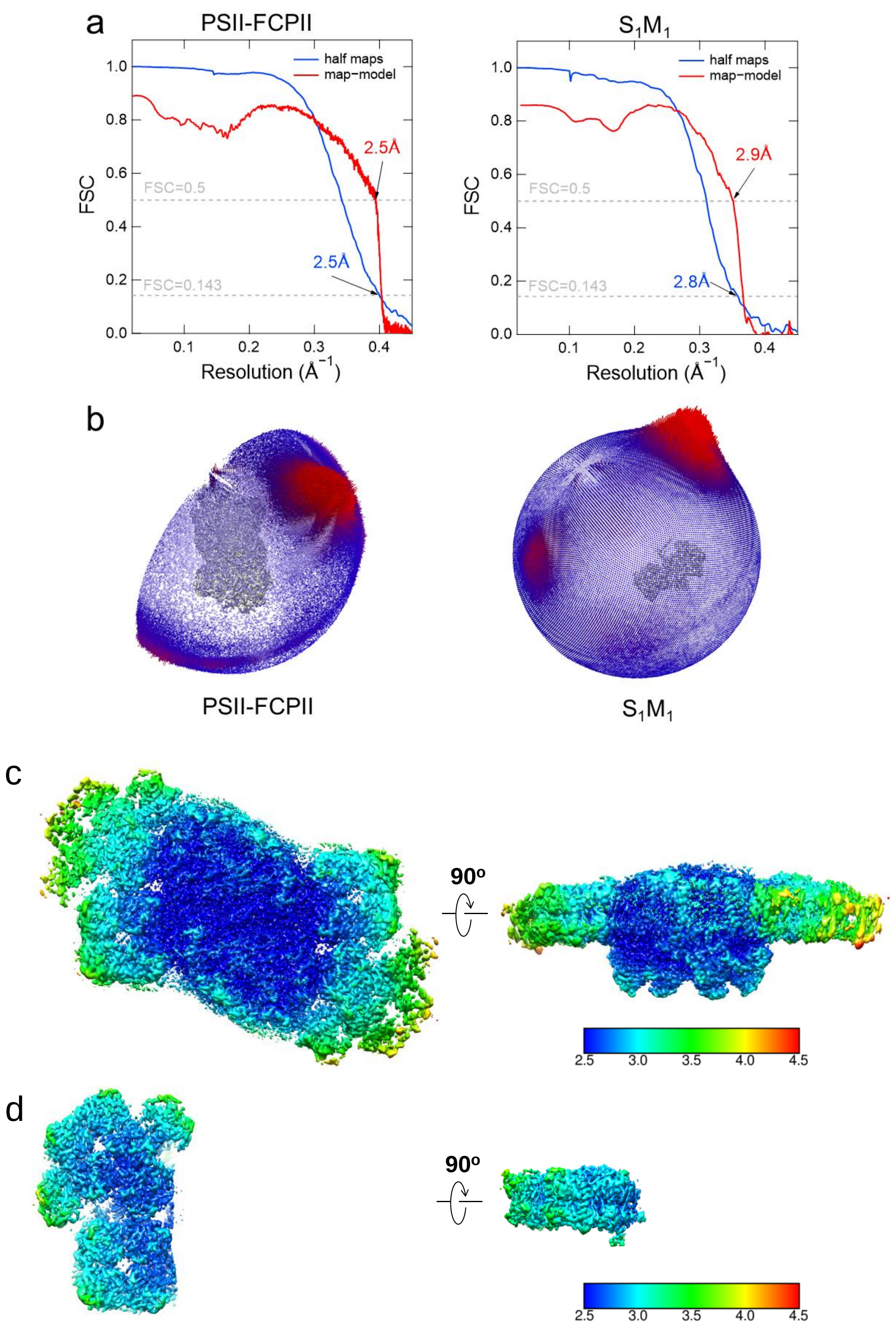

Supplementary Fig. 2. Evaluation of the cryo-EM map quality. a, FSC curves of PSII-FCPII ($C_2S_2M_2$) and S_1M_1 for independently refined half maps (blue) and for map-

minus-model (red). **b,** Angular distribution of the particles used to reconstruct the PSII-FCPII ($C_2S_2M_2$) and S_1M_1 structures. Each cylinder represents one view, and the height of the cylinder is proportional to the number of particles for that view. **c,** Local resolution maps of PSII-FCPII ($C_2S_2M_2$). **d,** Local resolution maps of S_1M_1.

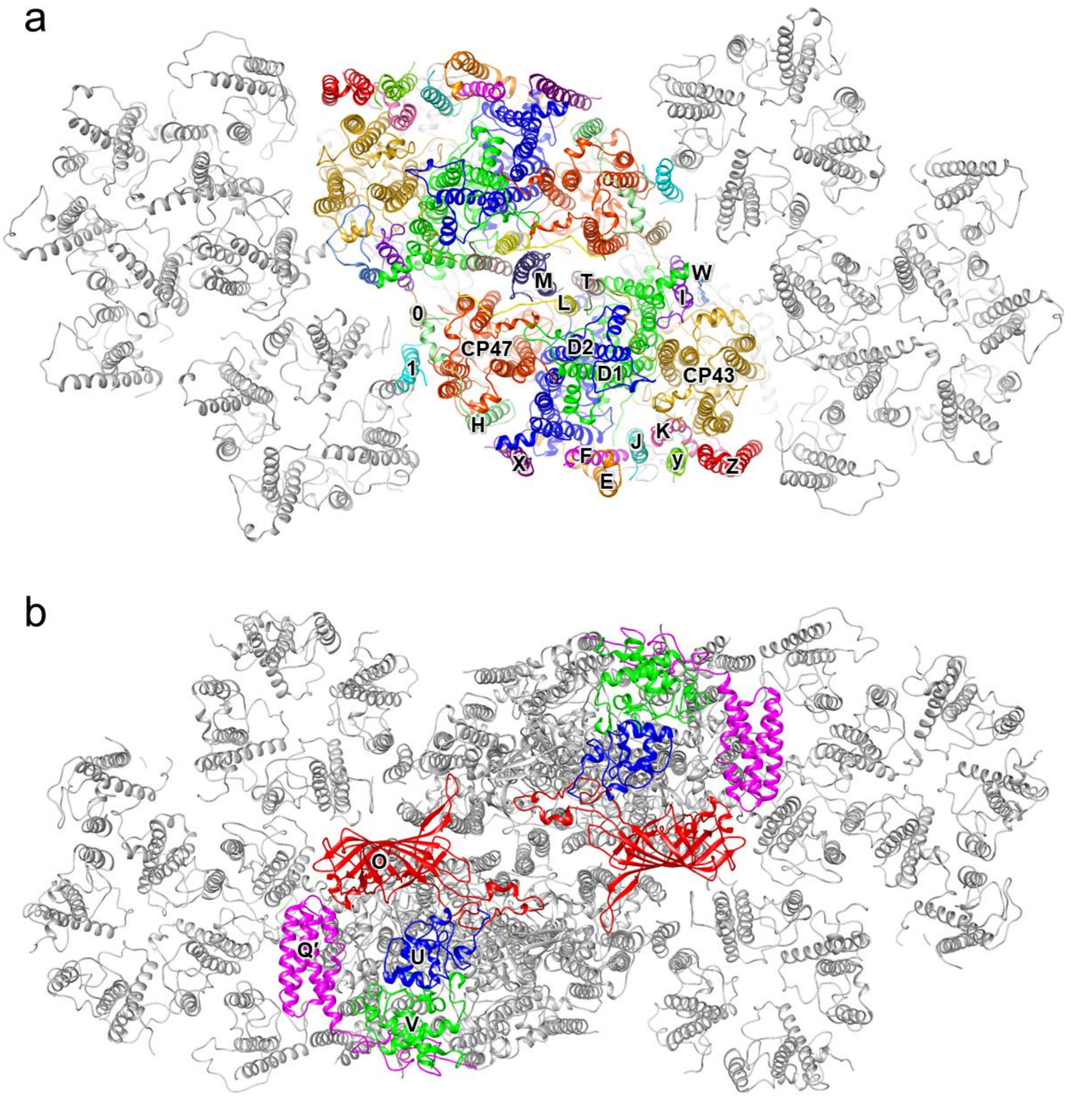

Supplementary Fig. 3. Structure of the PSII cores in the PSII-FCPII supercomplex. Overall structure of the PSII-FCPII supercomplex viewed from the stromal (**a**) and lumenal (**b**) sides. FCPII is colored gray, whereas PSII subunits are depicted in different colors indicated by D1, CP47, CP43, D2, PsbE (E), PsbF (F), PsbH (H), PsbI (I), PsbJ (J), PsbK (K), PsbL (L), PsbM (M), PsbT (T), PsbW (W), PsbX (X), ycf12 (y), PsbZ (Z), Unknown0 (0), Unknown1 (1), PsbO (O), PsbQ′ (Q′), PsbV (V), and PsbU (U).

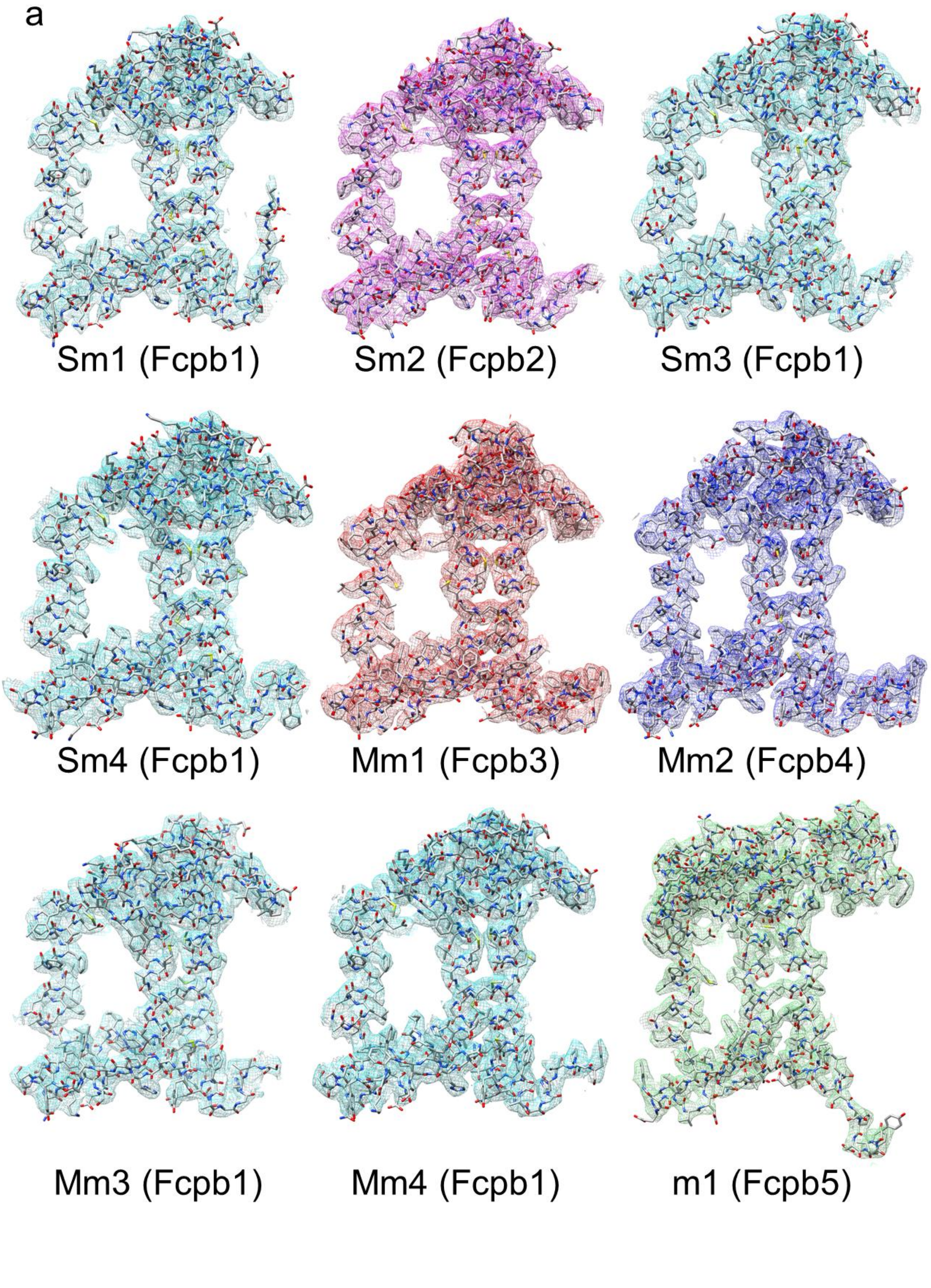
a
Sm1 (Fcpb1)
Sm2 (Fcpb2)
Sm3 (Fcpb1)
Sm4 (Fcpb1)
Mm1 (Fcpb3)
Mm2 (Fcpb4)
Mm3 (Fcpb1)
Mm4 (Fcpb1)
m1 (Fcpb5)

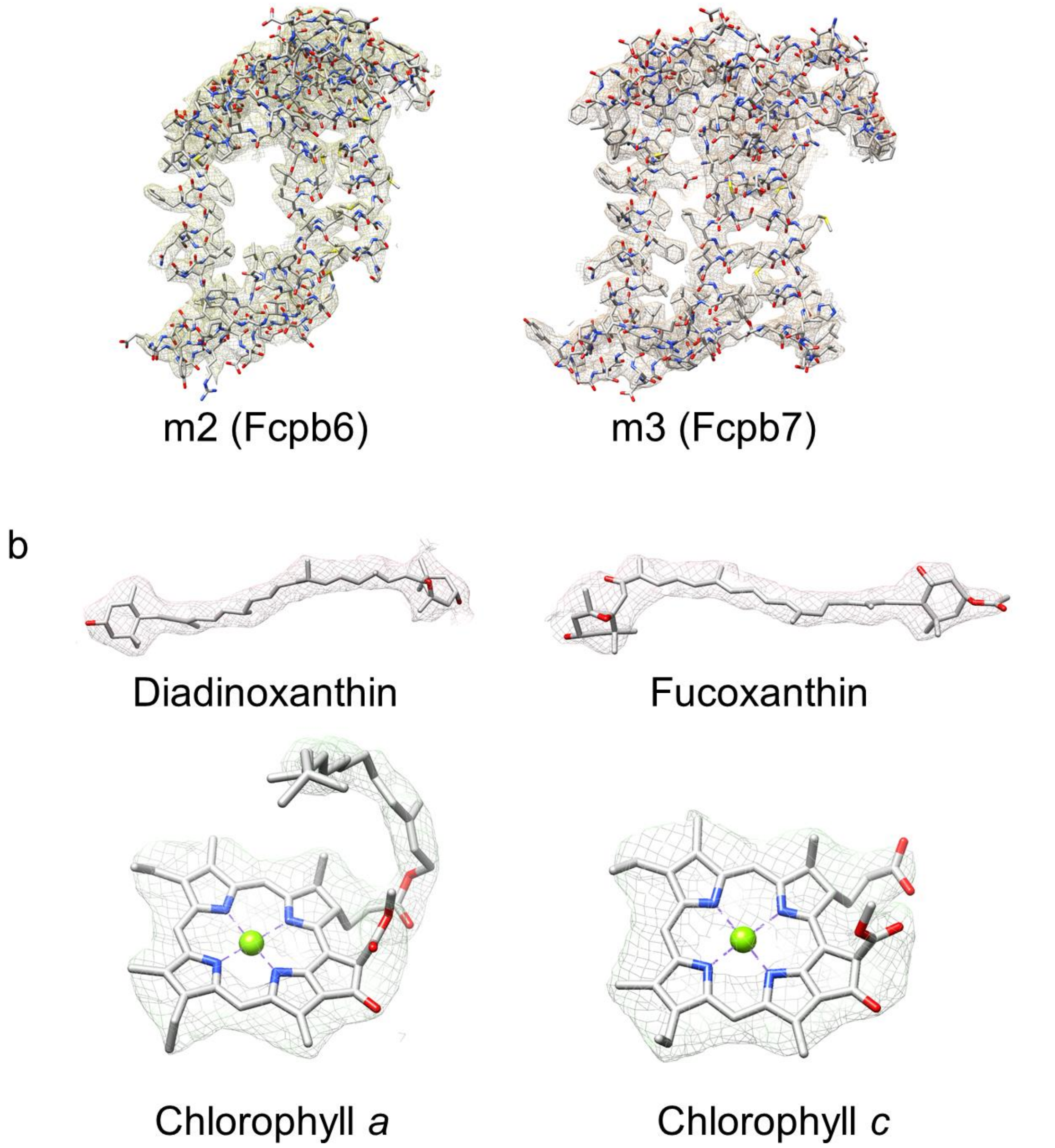

Supplementary Fig. 4. Cryo-EM density maps and structures of the FCPII subunits and characteristic pigment molecules. a, The cryo-EM density maps and corresponding models of FCPII subunits are shown as meshes and sticks, respectively. **b,** The cryo-EM density maps and corresponding models of representative pigment molecules are shown as meshes and sticks, respectively. Meshes are represented at 15 σ contour level using the cryo-EM map of S_1M_1.

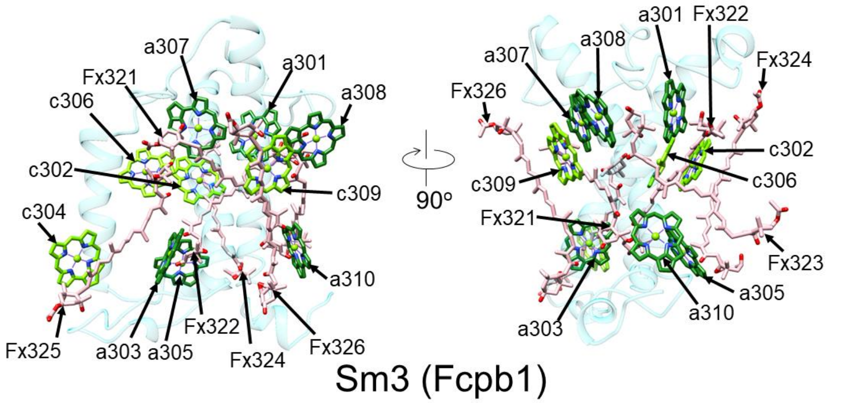

Sm3 (Fcpb1)

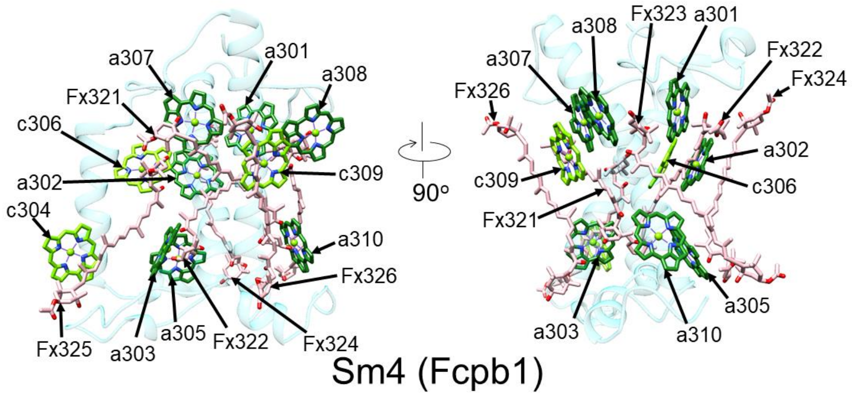

Sm4 (Fcpb1)

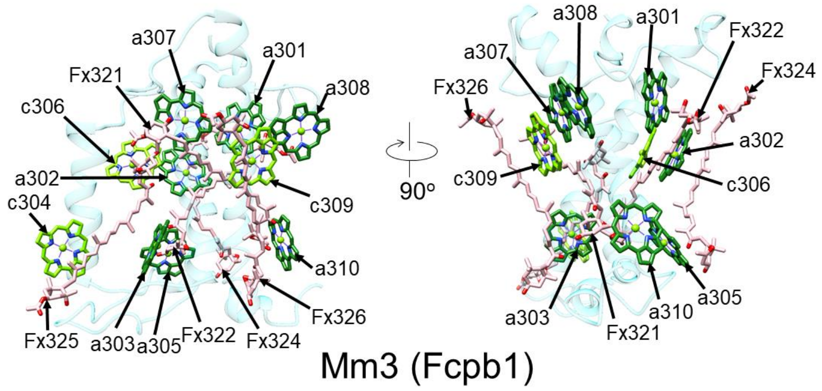

Mm3 (Fcpb1)

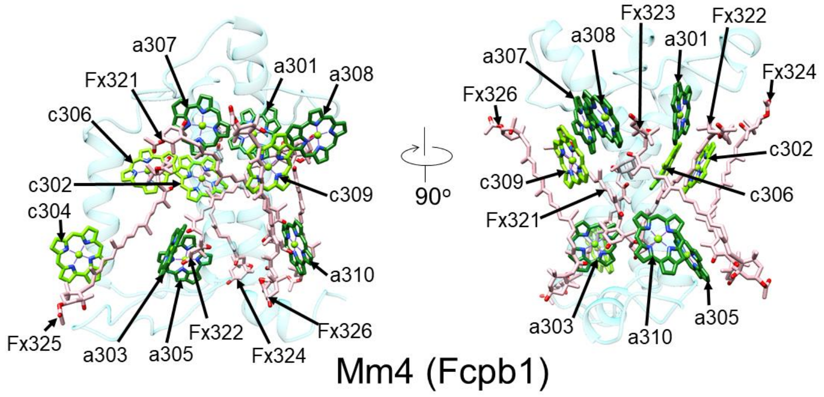

Mm4 (Fcpb1)

Supplementary Fig. 5. Structures of Sm3, Sm4, Mm3, and Mm4. Structures of Sm3-Fcpb1, Sm4-Fcpb1, Mm3-Fcpb1, and Mm4-Fcpb1 depicted with Cα atoms and arrangements of the pigments (Chls and Cars), with the right-side panel rotated 90° clockwise relative to the left-side panel in each subunit. The pigments of Chl *a*, Chl *c*, and Fx are colored “forest green”, “bright green”, and “pink”, respectively. Only rings of the Chls are depicted.

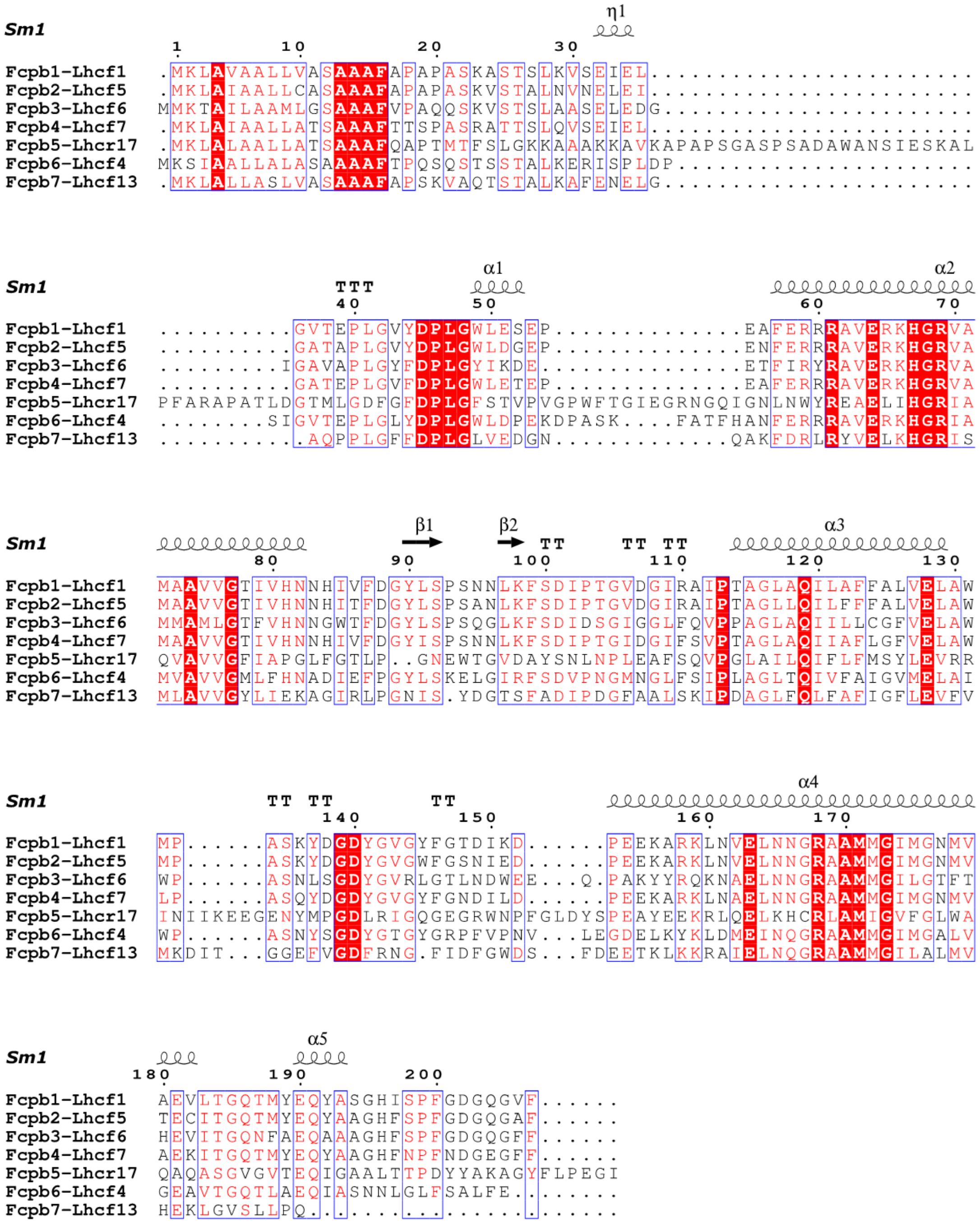

Supplementary Fig. 6. Comparison of amino-acid sequences among seven Fcpbs. Multiple sequence alignment was carried out using ClustalW [https://www.genome.jp/tools-bin/clustalw] and ESPript [http://espript.ibcp.fr/ESPript/ESPript/]. Secondary-structural elements of Sm1 are shown above the sequences. Completely conserved residues are highlighted in red.

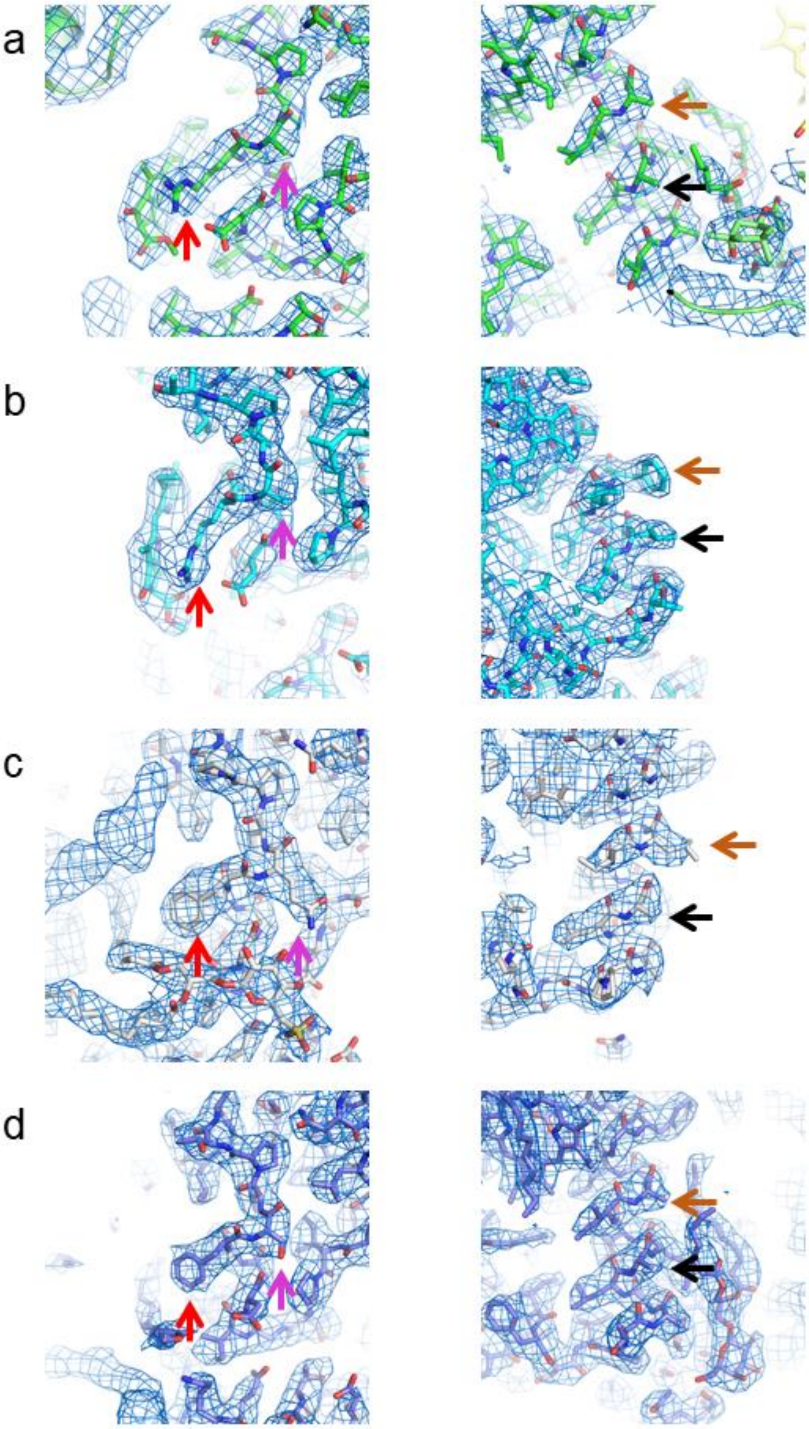

a
b
c
d

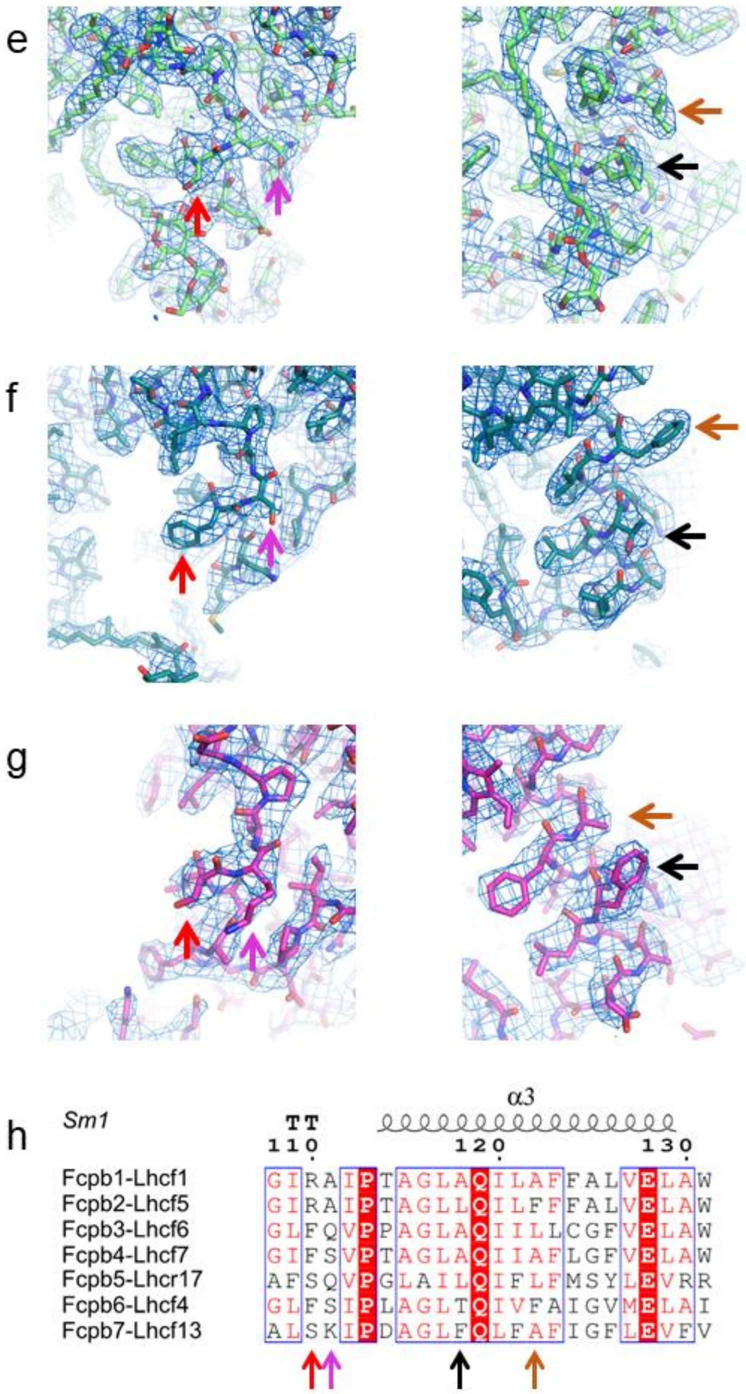

Supplementary Fig. 7. Characteristic amino-acid residues used for the identification of each Fcpb subunit. a–g, Characteristic maps and residues of Fcpb1 (**a**), Fcpb2 (**b**), Fcpb3 (**c**), Fcpb4 (**d**), Fcpb5 (**e**), Fcpb6 (**f**), and Fcpb7 (**g**). The maps are shown as meshes at 15 σ contour level using the cryo-EM map of S_1M_1, and the corresponding models are shown as sticks. Unique residues used for identification are indicated by red, magenta, black, and orange arrows, respectively. **h,** Amino-acid sequences corresponding to the characteristic residues.

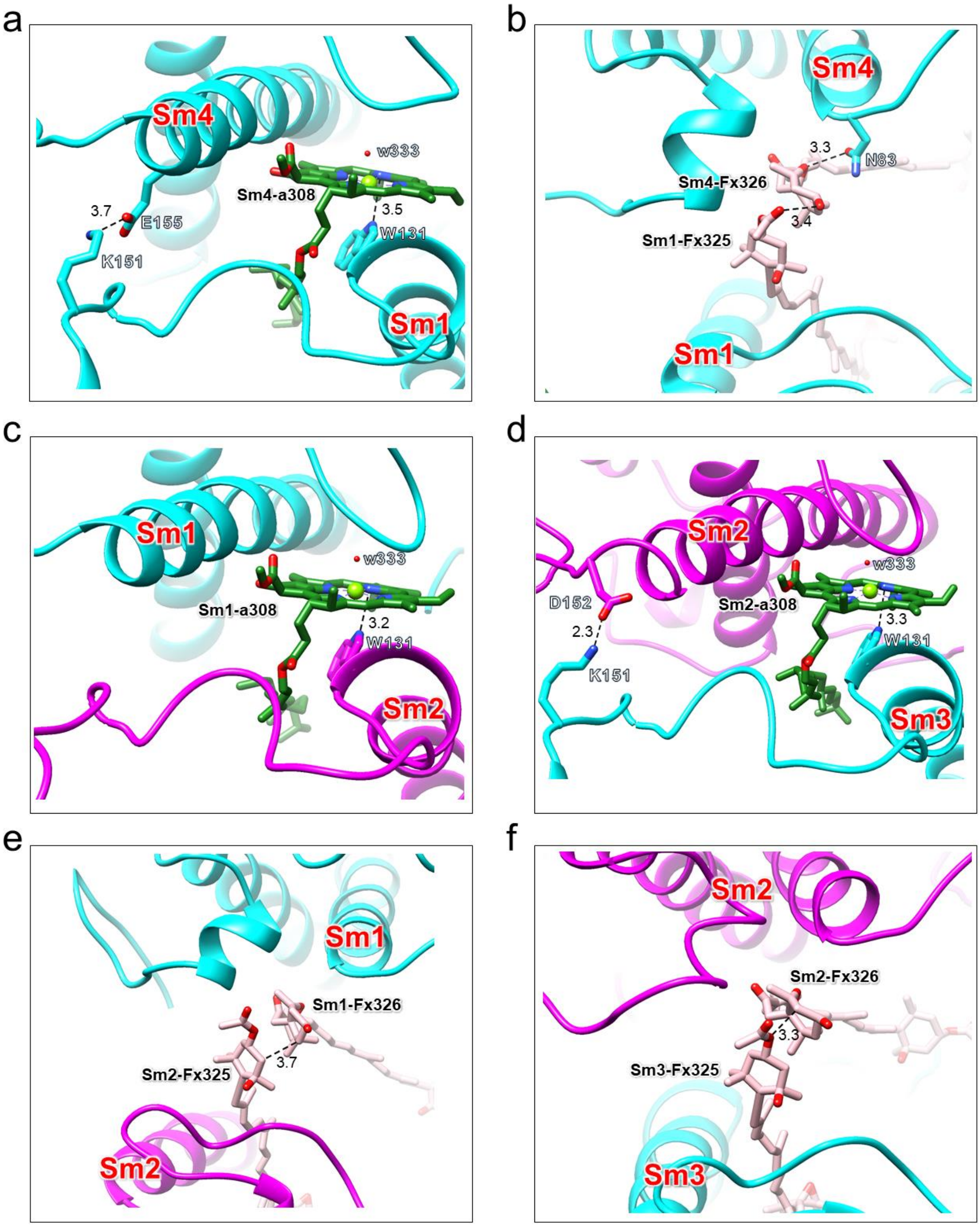
a
Sm4
w333
Sm4-a308
3.7
E155
3.5
W131
K151
Sm1
b
Sm4
3.3
N83
Sm4-Fx326
3.4
Sm1-Fx325
Sm1
c
Sm1
w333
Sm1-a308
3.2
W131
Sm2
d
Sm2
w333
D152
Sm2-a308
2.3
3.3
W131
K151
Sm3
e
Sm1
Sm1-Fx326
Sm2-Fx325
3.7
Sm2
f
Sm2
Sm2-Fx326
3.3
Sm3-Fx325
Sm3

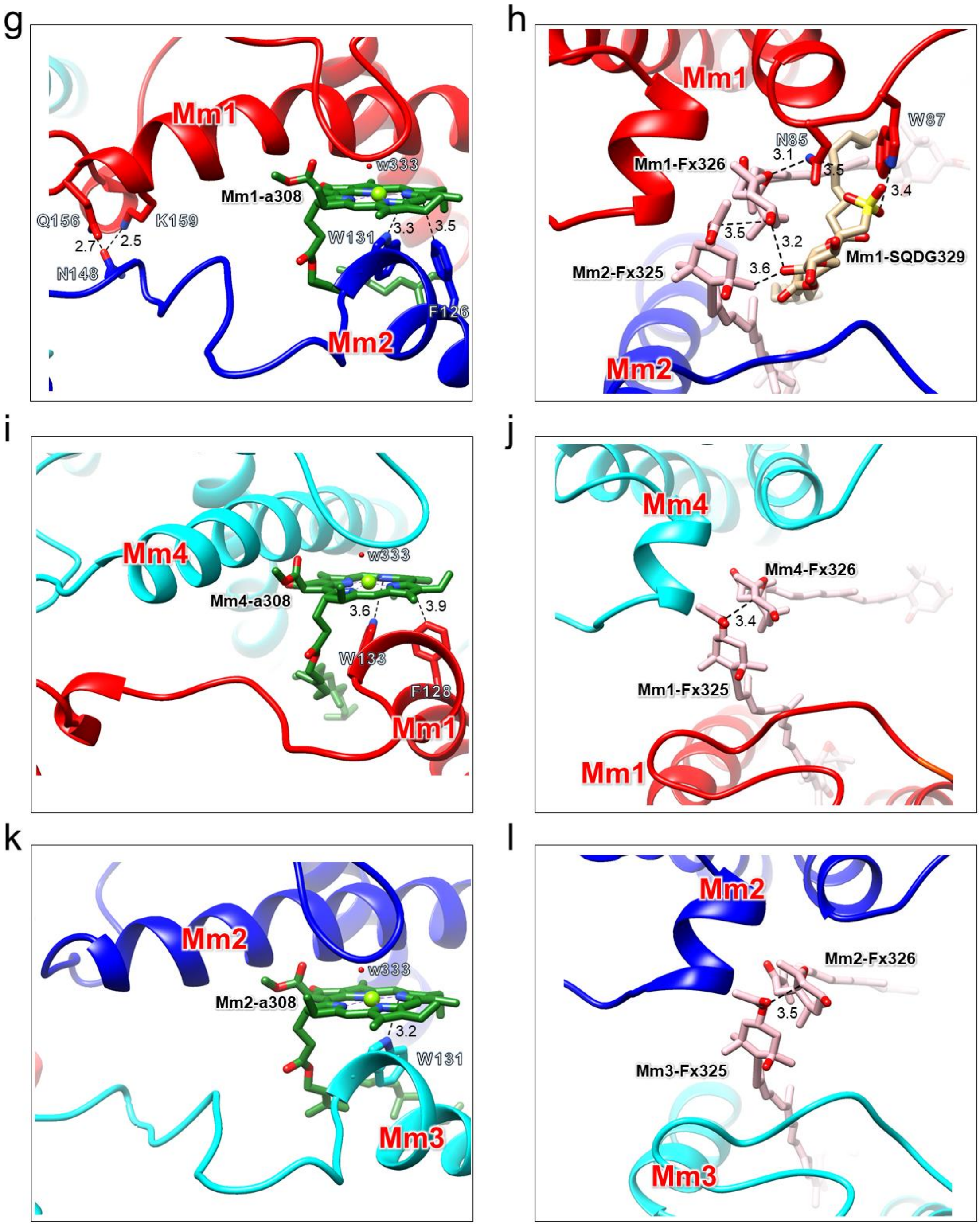
g
Mm1
w333
Mm1-a308
Q156
K159
2.7
2.5
N148
W131
3.3
3.5
F126
Mm2
h
Mm1
W87
N85
3.1
Mm1-Fx326
3.5
3.4
3.5
3.2
Mm1-SQDG329
Mm2-Fx325
3.6
Mm2
i
Mm4
w333
Mm4-a308
3.6
3.9
W133
F128
Mm1
j
Mm4
Mm4-Fx326
3.4
Mm1-Fx325
Mm1
k
Mm2
w333
Mm2-a308
3.2
W131
Mm3
l
Mm2
Mm2-Fx326
3.5
Mm3-Fx325
Mm3

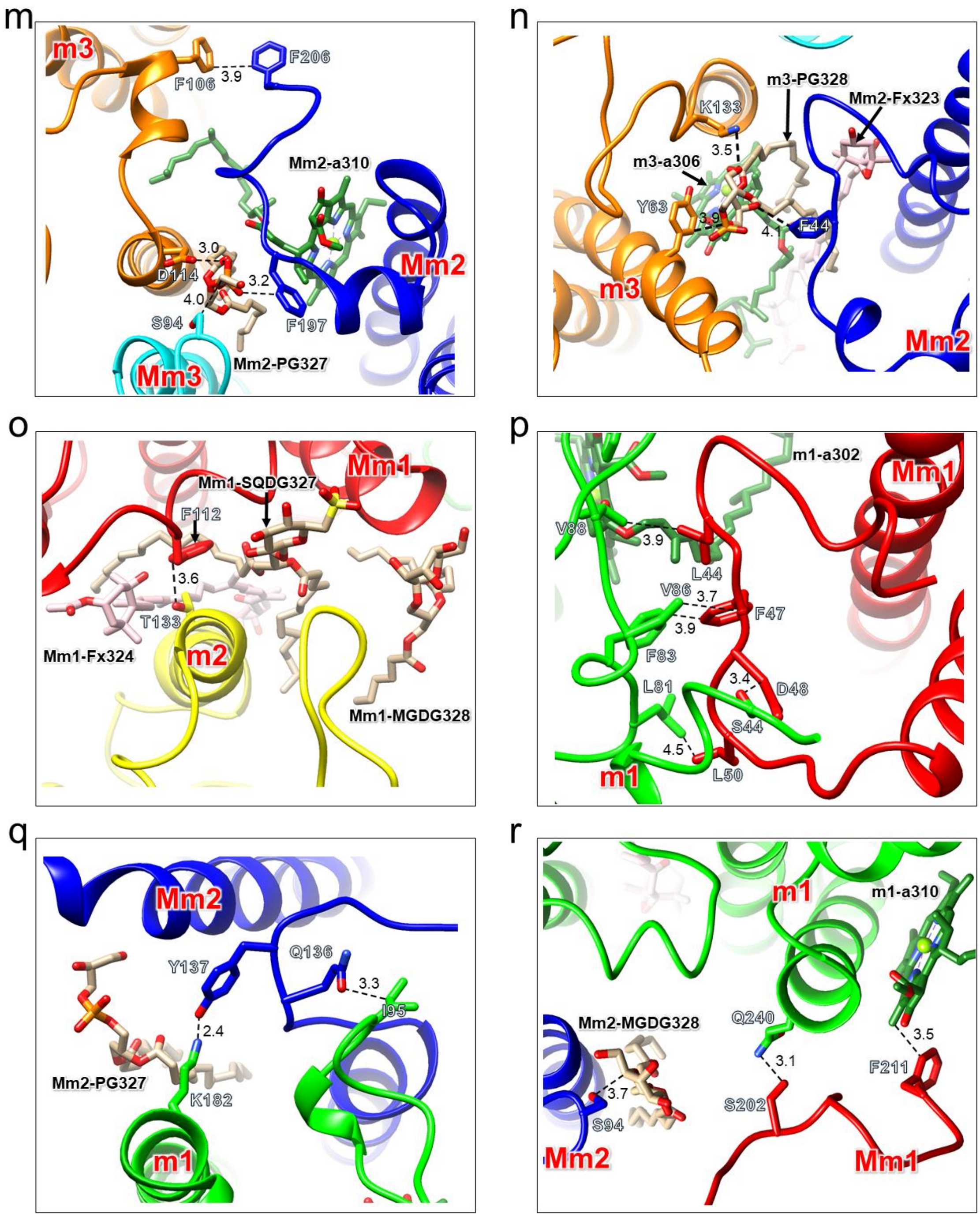

Supplementary Fig. 8. Interactions among the FCPII units. a, Interactions between Sm1 and Sm4 at the stromal side. **b,** Interactions between Sm1 and Sm4 at the lumenal side. **c,** Interactions between Sm1 and Sm2 at the stromal side. **d,** Interactions between Sm2 and Sm3 at the stromal side. **e,** Interactions between Sm1 and Sm2 at the lumenal side. **f,** Interactions between Sm2 and Sm3 at the lumenal side. **g,** Interactions between Mm1 and Mm2 at the stromal side. **h,** Interactions between Mm1 and Mm2 at the lumenal

side. **i,** Interactions between Mm1 and Mm4 at the stromal side. **j,** Interactions between Mm1 and Mm4 at the lumenal side. **k,** Interactions between Mm2 and Mm3 at the stromal side. **l,** Interactions between Mm2 and Mm3 at the lumenal side. **m,** Interactions among m3, Mm2, and Mm3 at the lumenal side. **n,** Interactions between m3 and Mm2 at the stromal side. **o,** Interactions between m2 and Mm1 at the lumenal side. **p,** Interactions between m1 and Mm1 at the stromal side. **q,** Interactions between m1 and Mm2 at the stromal side. **r,** Interactions among m1, Mm1, and Mm2 at the lumenal side. The pigments of Chl *a*, Chl *c*, Fx, and Ddx are colored "forest green", "bright green", "pink", and "purple", respectively. Only rings of the Chls are depicted. Interactions are indicated by dashed lines and numbers are distances in Å.

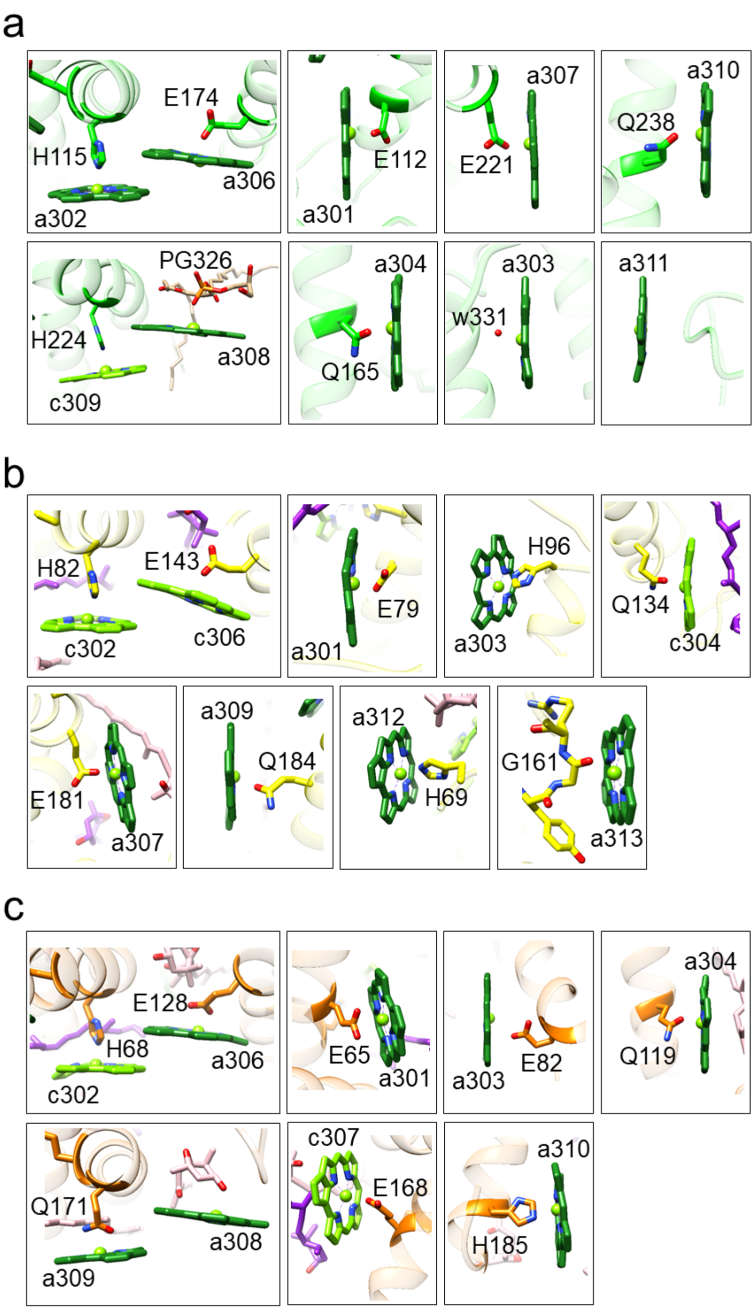

Supplementary Fig. 9. Interactions of Chls with ligands in m1 (a), m2 (b), and m3 (c). The pigments of Chl *a*, Chl *c*, Fx, and Ddx are colored “forest green”, “bright green”, “pink”, and “purple”, respectively. Only rings of the Chls are depicted.

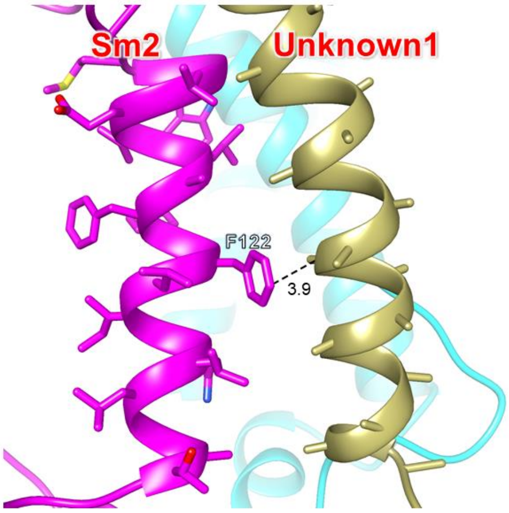

Supplementary Fig. 10. Interactions between Sm2 and Unknown1. The characteristic interaction between Sm2 and Unknown1 stands for a dashed line with a distance of 3.9 Å.

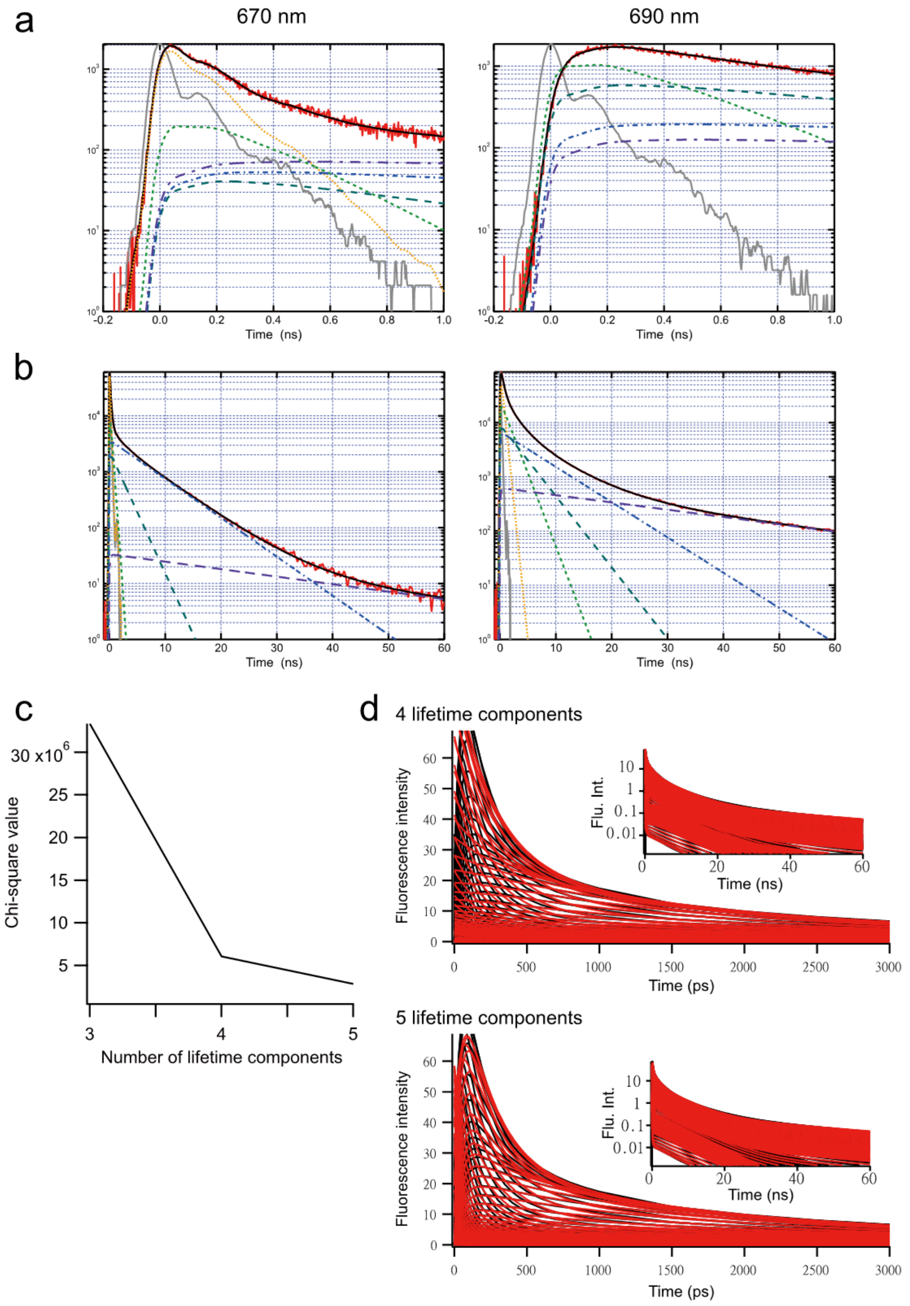

Supplementary Fig. 11. Relevance of global-fitting analysis. a,b, Fluorescence decay curves and their fitting results at 670 nm and 690 nm in time ranges until 1 ns (**a**) and 60 ns (**b**). Red lines, fluorescence decay curves; Gray lines, instrumental function; Black lines, fitting curves; dotted lines, components. **c,** Chi-square curves during global analysis. Large chi-square value means that the fitting is not good. According to these curves, the sample requires four or five lifetime components. **d,** Global-fitting results using four or five lifetime components. Black lines, fluorescence decay curves after deconvolution; red lines, fitting curves. In a shorter time region, the fitting is not good with the four lifetime components, so that we choose the five lifetime components.

Supplementary Table 1. Cryo-EM data collection and structural analysis statistics.

Complex	$C_2S_2M_2$	S_1M_1
PDB ID	7VD5	7VD6
EMDB ID	EMD-31905	EMD-31906
Data collection and processing		
Magnification	59000	59000
Voltage (kV)	300	300
Electron exposure (e⁻/Å)	50	50
Defocus range (μm)	−1.5 to −3.5	−1.5 to −3.5
Pixel size (Å)	1.113	1.113
Symmetry imposed	C2	C1
Final particle images (no.)	210825	373897
Map resolution (Å)	2.5	2.8
FSC threshold	0.143	0.143
Refinement		
Initial Model used (PDB code)	6J40	6J40
Model resolution (Å)	2.5	2.9
FSC threshold	0.5	0.5
Map sharpening B factor ($Å^2$)	−70	−83
Model composition		
Non-hydrogen atoms	100894	23937
Protein	73942	14745
Ligand	26900	9179
Water	52	13
B factors ($Å^2$)		
Protein	40.8	63.7
Ligand	56.7	59.5
Water	36.5	51.7
R.m.s deviations		
Bond lengths (Å)	0.021	0.026
Bond angles (°)	2.23	2.41
Validation		
MolProbity score	1.96	1.72
Clashscore	4.95	5.81
Poor rotamers (%)	3.00	0.27
EMRinger score	3.59	3.72
Ramachandran plot		
Favored (%)	95.15	93.98
Allowed (%)	4.79	5.86
Disallowed (%)	0.06	0.16

Supplementary Table 2. Averaged Q-score in each subunit.

Subunit	Averaged Q-score	
	$C_2S_2M_2$	S_1M_1
D1	0.78	-
CP47	0.77	-
CP43	0.77	-
D2	0.78	-
PsbE	0.72	-
PsbF	0.71	-
PsbH	0.75	-
PsbI	0.77	-
PsbJ	0.75	-
PsbK	0.75	-
PsbL	0.77	-
PsbM	0.75	-
PsbO	0.72	-
PsbT	0.77	-
PsbU	0.72	-
PsbV	0.73	-
PsbW	0.69	-
PsbX	0.66	-
ycf12	0.70	-
PsbZ	0.70	-
PsbQ′	0.71	-
Unknown1	0.73	-
Unknown2	0.72	-
Sm1	0.71	0.70
Sm2	0.69	0.68
Sm3	0.55	0.64
Sm4	0.57	0.66
Mm1	0.56	0.71
Mm2	0.52	0.70
Mm3	0.34	0.66
Mm4	0.34	0.66
m1	0.65	0.71
m2	0.41	0.62
m3	0.31	0.62

Supplementary Table 3. Cofactors in each monomer unit of the PSII-FCPII supercomplex.

Protein	Chlorophyll	Carotenoid	Heme	Lipid	Others
D1 (PsbA)	4 Chl *a*	1 BCR	-	1 SQD<br>1 LMG	1 Mn_4CaO_5 cluster,<br>1 plastoquinone,<br>2 pheophytin,<br>1 non-heme iron
CP47 (PsbB)	16 Chl *a*	3 BCR	-	2 SQD<br>2 LMG	-
CP43 (PsbC)	13 Chl *a*	2 BCR	-	3 DGD<br>1 LMG	-
D2 (PsbD)	2 Chl *a*	1 BCR	-	3 LHG<br>1 LMG	1 plastoquinone,<br>1 bicarbonate ion
PsbF	-	-	1	-	-
PsbH	-	1 BCR	-	1 DGD	-
PsbK	-	2 BCR	-	-	-
PsbL	-	-	-	1 LHG	-
PsbM	-	-	-	1 LMG	-
PsbT	-	1 BCR	-	-	-
PsbV	-	-	1	-	-
PsbZ	1 Chl *a*	-	-	-	-
PsbW	2 Chl *a*	-	-	-	-
Unknown1	1 Chl *a*	1 BCR	-	1 LMG	-
Sm1 (Fcpb1)	6 Chl *a,* 4 Chl *c*	6 Fx	-	2 LMG	-
Sm2 (Fcpb2)	7 Chl *a,* 3 Chl *c*	6 Fx	-	-	-
Sm3 (Fcpb1)	6 Chl *a,* 4 Chl *c*	6 Fx	-	-	-
Sm4 (Fcpb1)	7 Chl *a,* 3 Chl *c*	6 Fx	-	-	-
Mm1 (Fcpb3)	5 Chl *a,* 4 Chl *c*	5 Fx, 1 Ddx	-	1 LMG<br>2 SQD	-
Mm2 (Fcpb4)	5 Chl *a,* 4 Chl *c*	6 Fx	-	1 LHG<br>1 LMG	-
Mm3 (Fcpb1)	7 Chl *a,* 3 Chl *c*	5 Fx	-	1 LHG	-
Mm4 (Fcpb1)	6 Chl *a,* 4 Chl *c*	6 Fx	-		-
m1 (Fcpb5)	9 Chl *a,* 1 Chl *c*	2 Fx, 1 Ddx	-	1 LHG	-
m2 (Fcpb6)	6 Chl *a,* 3 Chl *c*	2 Fx, 2 Ddx	-		-
m3 (Fcpb7)	7 Chl *a,* 2 Chl *c*	4 Fx, 2 Ddx	-	1 LHG	-
Total	145	72	2	28	7

BCR, *β*-carotene; SQD, sulfoquinovosyldiacyl glycerol; LMG, distearoylmonogalactosyl diglyceride; DGD, digalactosyldiacyl glycerol; LHG, dipalmitoylphosphatidyl glycerol; Fx, fucoxanthin; Ddx, diadinoxanthin; Chl *a*, chlorophyll *a*; Chl *c*, chlorophyll *c*.

Supplementary Table 4. Fcpb proteins identified in the PSII-FCPII structure.

Protein	Gene	Gene ID[a]	Identity (%)[b]	Similarity (%)[b]	RMSD (Å) / Aligned Cα atoms
Fcpb1 (Sm1)	*Lhcf1*	LC440426 /g14370	100	100	0.00 / 176
Fcpb2 (Sm2)	*Lhcf5*	g2935	87	93	0.42 / 169
Fcpb1 (Sm3)	*Lhcf1*	LC440426 /g14370	100	100	0.43 / 169
Fcpb1 (Sm4)	*Lhcf1*	LC440426 /g14370	100	100	0.44 / 171
Fcpb3 (Mm1)	*Lhcf6*	g5499	59	69	1.07 / 162
Fcpb4 (Mm2)	*Lhcf7*	g6797	84	92	0.50 / 169
Fcpb1 (Mm3)	*Lhcf1*	LC440426 /g14370	100	100	0.45 / 168
Fcpb1 (Mm4)	*Lhcf1*	LC440426 /g14370	100	100	0.47 / 169
Fcpb5 (m1)	*Lhcr17*	g3978	26	33	2.06 / 139
Fcpb6 (m2)	*Lhcf4*	g2721	49	64	0.93 / 134
Fcpb7 (m3)	*Lhcf13*	LC647435[c]	43	56	1.56 / 143

[a]Accession No. in the DDBJ/EMBL/GenBank databases and/or Gene ID in ChaetoBase v1.1, the gene annotation database for *Chaetoceros gracilis* (https://chaetoceros.nibb.ac.jp).
[b]Identity and similarity between the amino acid sequence of Fcpb1 and those of other Fcpbs were estimated by an optimal global alignment of two sequences using EMBOSS needle (https://www.ebi.ac.uk/Tools/psa/emboss_needle/) with BLOSUM62 matrix and default settings.
[c]*Lhcf13* was identified in the transcriptome data but not in the draft genome assembly.

Supplementary Table 5. Correspondence of numbering of pigments in the S- and M-tetramers described in the text with those in the PDB file of the PSII-FCPII supercomplex.

FCPs	Sm1	Sm2	Sm3	Sm4
**Chls in the text**	**PDB No. (Chain ID)**	**PDB No. (Chain ID)**	**PDB No. (Chain ID)**	**PDB No. (Chain ID)**
**301**	301 (11), 302 (31)	203 (12), 204 (32)	301 (13, 33)	301 (14, 34)
**302**	302 (11), 303 (31)	204 (12), 205 (32)	302 (13, 33)	302 (14, 34)
**303**	303 (11), 304 (31)	205 (12), 206 (32)	303 (13, 33)	303 (14, 34)
**304**	304 (11), 305 (31)	206 (12), 207 (32)	304 (13, 33)	304 (14, 34)
**305**	305 (11), 306 (31)	207 (12), 208 (32)	305 (13, 33)	305 (14, 34)
**306**	306 (11), 307 (31)	208 (12), 209 (32)	306 (13, 33)	306 (14, 34)
**307**	307 (11), 308 (31)	209 (12), 210 (32)	307 (13, 33)	307 (14, 34)
**308**	308 (11), 202 (32)	210 (12), 211 (32)	308 (13, 33)	308 (14, 34)
**309**	309 (11, 31)	211 (12), 212 (32)	309 (13, 33)	309 (14, 34)
**310**	310 (11, 31)	212 (12), 213 (32)	310 (13, 33)	310 (14, 34)
**Cars in the text**				
**321**	311 (11, 31)	213 (12), 214 (32)	311 (13, 33)	311 (14, 34)
**322**	312 (11, 31)	214 (12), 215 (32)	312 (13, 33)	312 (14, 34)
**323**	313 (11, 31)	215 (12), 216 (32)	313 (13, 33)	313 (14, 34)
**324**	314 (11, 31)	216 (12), 217 (32)	314 (13, 33)	314 (14, 34)
**325**	315 (11, 31)	319 (11, 31)	220 (12), 221(32)	315 (14, 34)
**326**	202 (12), 203 (32)	217 (12), 218 (32)	315 (13, 33)	316 (14, 34)

FCPs	Mm1	Mm2	Mm3	Mm4
**Chls in the text**	**PDB No. (Chain ID)**	**PDB No. (Chain ID)**	**PDB No. (Chain ID)**	**PDB No. (Chain ID)**
**301**	301 (16, 36)	302 (17), 301 (37)	202 (18, 38)	301 (15, 35)
**302**	302 (16, 36)	303 (17), 302 (37)	203 (18, 38)	302 (15, 35)
**303**	303 (16, 36)	304 (17), 303 (37)	204 (18, 38)	303 (15, 35)
**304**	304 (16, 36)	305 (17), 304 (37)	205 (18, 38)	304 (15, 35)
**305**	-	-	206 (18, 38)	305 (15, 35)
**306**	305 (16, 36)	306 (17), 305 (37)	207 (18, 38)	306 (15, 35)
**307**	306 (16, 36)	307 (17), 306 (37)	208 (18, 38)	307 (15, 35)
**308**	307 (16, 36)	201 (18, 38)	209 (18, 38)	308 (15, 35)
**309**	308 (16, 36)	308 (17), 307 (37)	210 (18, 38)	309 (15, 35)
**310**	309 (16, 36)	309 (17), 308 (37)	211 (18, 38)	310 (15, 35)
**Cars in the text**				
**321**	310 (16, 36)	310 (17), 309 (37)	212 (18, 38)	311 (15, 35)
**322**	311 (16, 36)	311 (17), 310 (37)	213 (18, 38)	312 (15, 35)
**323**	312 (16, 36)	312 (17), 311 (37)	-	313 (15, 35)
**324**	313 (16), 201 (40)	313 (17), 312 (37)	214 (18, 38)	314 (15, 35)
**325**	319 (15, 35)	314 (17), 313 (37)	320 (17), 319 (37)	315 (15, 35)
**326**	314 (16), 313 (36)	315 (17), 314 (37)	215 (18, 38)	316 (15, 35)

Supplementary Table 6. Correspondence of numbering of pigments in the FCP monomers described in the text with those in the PDB file of the PSII-FCPII supercomplex.

FCPs	m1	m2	m3
**Chls in the text**	**PDB No. (Chain ID)**	**PDB No. (Chain ID)**	**PDB No. (Chain ID)**
**301**	301 (19, 39)	301 (20), 203 (40)	202 (21, 41)
**302**	302 (19, 39)	302 (20), 204 (40)	203 (21, 41)
**303**	303 (19, 39)	303 (20), 205 (40)	204 (21, 41)
**304**	304 (19, 39)	304 (20), 206 (40)	205 (21, 41)
**306**	305 (19, 39)	305 (20), 207 (40)	206 (21, 41)
**307**	306 (19, 39)	306 (20), 208 (40)	207 (21, 41)
**308**	307 (19, 39)	-	208 (21, 41)
**309**	308 (19, 39)	307 (20), 209 (40)	209 (21, 41)
**310**	309 (19, 39)	-	210 (21, 41)
**311**	310 (19, 39)	-	-
**312**	-	308 (20), 210 (40)	-
**313**	-	102 (Z, z)	-
**Cars in the text**			
**321**	311 (19, 39)	309 (20), 211 (40)	211 (21, 41)
**322**	312 (19, 39)	310 (20), 212 (40)	212 (21, 41)
**323**	-	-	213 (21, 41)
**324**	-	311 (20), 213 (40)	214 (21, 41)
**325**	313 (19, 39)	312 (20), 214 (40)	215 (21, 41)
**327**	-	-	216 (21, 41)
